# 

**Published:** 1832-03

**Authors:** 


### MONTHLY LIST OF MEDICAL BOOKS.

[*Medical Works cannot be entered on this List unless a copy be sent for the purpose, the titles of Books having frequently been sent to us as published which have not been published for weeks, or even months, after.*]

Suggestions on the best Means of Supplying Anatomical Schools with Opportunity of Studying Practical Anatomy and Surgery. In a Letter addressed to the Right Hon. Viscount Melbourne, Secretary of State for the Home Department. By J. Evans Riadore, Esq., F.L.S., &c.

Published by Authority of the Lords of his Majesty's most Honourable Privy Council. Official Reports made to Government by Drs. Russel and Barry, on the Disease called Cholera Spasmodica, as observed by them during their Mission to Russia in 1831. With an Appendix and other Papers, Extracts of Letters, Reports, and Communications received from the Continent, relating to that Disease.—8vo. pp. 147. Winchester and Varnam, London.

*** No practitioner will be justified in remaining without these very valuable Reports.

Atlas of Surgical Apparatus; being a Series of Delineations of the most important Mechanical Auxiliaries of Surgery, with descriptive Letter-press, explaining their several Uses and Modes of Application. By Henry T. Chapman, Member of the Royal College of Surgeons, and late House Surgeon to St. Bartholomew's Hospital.—Twenty Plates. Highley.

A Brief Description of Surgical Apparatus; intended to accompany a Series of Delineations of the most important Mechanical Auxiliaries of Surgery. By Henry T. Chapman, Member of the Royal College of Surgeons, and late House Surgeon to St. Bartholomew's Hospital.—8vo. pp. 115. Highley, London.

Part IV. The Principles and Practice of Obstetric Medicine, in a Series of Systematic Dissertations on Midwifery, and on the Diseases of Women and Children. Illustrated by numerous Plates. By David D. Davis, M.D., M.R.S.L., Professor of Midwifery in the University of London, &c.—John Taylor, London.

An Account of the Beulah Saline Spa at Norwood, Surrey; containing a Description of its Medicinal Properties and Effects, of the Diseases in which it is remedial, and Directions for its Use. By George Hume Weatherhead, M.D., Member of the Royal College of Physicians, London, &c.—8vo. pp. 39. Hatchard, London.

A Modern Anatomy of the Human Spleen; to which are added, some Strictures on Sir Anthony Carlisle's Theory respecting the Functions of that Organ: addressed to the Medical Students and junior Practitioners of London. By P. Mac Nolty, Surgeon, &c.—8vo. pp. 71. Renshaw and Rush, London.

A Letter addressed to the Lord Chancellor, on the Study of Anatomy, &c. By J. C. SOMERVILLE, M.D.—Hatchard, London.

Examination Questions on Surgery and the Practice of Physic, for the Use of Students. By Sir CHARLES ALDIS, Surgeon and Accoucheur, &c.—12mo. Highley, London.

*** A work which any man might conceive, commence, and complete in an hour, if he had so much time to throw away upon such a worthless labour: it consists of questions in surgery and physic, without answers.

Substance of a Lecture on Cholera, read before the Medical Profession of Liverpool, on Wednesday, January 18, 1832. By D. BAIRD, M.D.—8vo. pp. 45. Liverpool and London.

The Laws and Progress of the Epidemic Cholera, illustrated by Facts and Observations. By THOMAS HANCOCK, M.D.—8vo. pp. 118. Hamilton and Adams, London.

(Part II. February 1832.) The Cyclopædia of Practical Medicine. Edited by JOHN FORBES, M.D., A. TWEEDIE, M.D., and JOHN CONOLLY, M.D.—Royal 8vo. pp. 224. Sherwood and Co. London.

On the Portable Sudatory, or Hot-air Bath; with Cases illustrative of its Medical Powers in various Disorders, and its great Utility in Cholera Morbus, with Directions for its Administration; together with Remarks on the Applicability of Galvanism in the first Stage of that Disease. By M. LA BEAUME, Medical Galvanist and Electrician in Ordinary to the King, &c. Second Edition.—12mo. Highley, London.

*** This edition of Mr. La Beaume's work contains much additional evidence of the efficacy of the "Sudatory" in cholera and various other diseases, together with some interesting remarks on the applicability of galvanism in the first stage of cholera.

*** *Communications have been received from* DR. ROBERTS, *&c.*

